# Affinity of Electrochemically Deposited Sol–Gel Silica Films towards Catecholamine Neurotransmitters

**DOI:** 10.3390/s19040868

**Published:** 2019-02-19

**Authors:** María Porcel-Valenzuela, Francisco Huerta, Emilia Morallón, Francisco Montilla

**Affiliations:** 1 Departmento Química Física e Instituto Universitario de Materiales, Universidad de Alicante, Carretera San Vicente del Raspeig s/n San Vicente del Raspeig, E-03690 Alicante, Spain; maria.porcel@ua.es (M.P.-V.); morallon@ua.es (E.M.); 2 Departmento Ingenieria Textil y Papelera, Universitat Politecnica de Valencia, Plaza Ferrandiz y Carbonell 1, E-03801 Alcoy, Spain; frahuear@txp.upv.es

**Keywords:** electroassisted deposition, catecholamine neurotransmitters, liquid adsorption isotherms

## Abstract

Dopamine, norepinephrine, and epinephrine neurotransmitters can be detected by electrochemical oxidation in conventional electrodes. However, their similar chemical structure and electrochemical behavior makes a difficult selective analysis. In the present work, glassy carbon electrodes have been modified with silica layers, which were prepared by electroassisted deposition of sol–gel precursors. These layers were morphologically and compositionally characterized using different techniques, such as field emission scanning electron microscopy (FESEM), TEM, FTIR, or thermogravimetric analysis–mass spectrometry (TG-MS). The affinity of silica for neurotransmitters was evaluated, exclusively, by means of electrochemical methods. It was demonstrated that silica adsorbs dopamine, norepinephrine, and epinephrine, showing different interaction with silica pores. The adsorption process is dominated by a hydrogen bond between silanol groups located at the silica surface and the amine groups of neurotransmitters. Because of the different interaction with neurotransmitters, electrodes modified with silica films could be used in electrochemical sensors for the selective detection of such molecules.

## 1. Introduction

Identification and quantification of catecholamine neurotransmitters (NT), dopamine (DA), epinephrine (EP), and norepinephrine (NE) in physiological fluids are key factors for an appropriate diagnosis of several neurodegenerative diseases and mental disorders [1]. Particularly, altered levels of these compounds can be found in schizophrenia, Parkinson’s disease, Alzheimer’s disease, or depression, among others [2,3,4]. Therefore, monitoring the concentration of these compounds could be a suitable strategy for the early detection of those diseases and a number of analytical methods have been used for such purpose: so far spectrophotometry, electrochemical detection, immunoassays, and liquid chromatography [5,6,7]. Particularly in the latter technique, the mobile phase (usually a mixture acetonitrile–water) passes through a column whose filler is commonly made of chemically modified silica [7,8,9]. However, chromatography is an expensive, complex analytical tool that requires lengthy sample treatments for a proper detection. Since catecholamine neurotransmitters are electroactive molecules, it would be desirable to develop a direct electrochemical sensor able to overcome several of the problems presented by the aforementioned analytical methods. However, electrochemical detection faces up to low selectivity because these NT show akin chemical structures and their oxidation takes place at fairly close potentials. Additionally, in physiological fluids, the presence of other electroactive species (like ascorbic or uric acids) at much higher concentration masks the redox reactions of neurotransmitters, which constitutes an additional complication that needs to be resolved.

It is widely-accepted that a suitable strategy to improve the selectivity of electrochemical detection could involve the use of modified electrodes able to interact more specifically with neurotransmitters. For this purpose it has been reported the use of polypyrrole films [10], nickel oxide nanoparticles, or carbon nanotubes within dihexadecylphosphate films [11], as well as carbon-modified electrodes [12,13]. To our knowledge, the use of silica-based materials for neurotransmitter detection has been restricted to their integration with carbon nanoparticles to enhance sensitivity [14]. Despite its dielectric behavior, silica obtained by sol–gel methodologies demonstrated unique properties when used as active electrode components [15,16,17]. In fact, silica films exhibit modulated permselectivity and ion-exchange ability, making it possible to discern between positively and negatively charged probes [18]. In addition, silica films can be molecularly imprinted with the target species to improve the analytical response. Particularly, it has been reported that the molecular impression with dopamine leads to a modified material showing high selectivity for this species, even in the presence of highly concentrated ascorbic acid (dopamine molar ratio lower than 1:50,000) [19].

In this work we focus, on the one hand, on the morphological and compositional characterization of silica films prepared by electrochemical methods from sol–gel precursors and, on the other, on their application as selective sensors for catecholamine neurotransmitters. The physicochemical nature of the interaction between these molecules and the silica surface will be characterized by applying a thermodynamic analysis to the electrochemical results.

## 2. Experimental

The reagents used were tetraethyl orthosilicate (TEOS, Sigma-Aldrich, St. Louis, MO, USA, reagent grade), dopamine hydrochloride (DA, Sigma-Aldrich), (-)-epinephrine (EP, Sigma-Aldrich, reagent grade), (-)-norepinephrine (NE, Sigma-Aldrich, reagent grade), potassium dihydrogen phosphate (Merck, p.a.), potassium hydrogen phosphate (Merck, p.a.), ethanol (EtOH, Sigma Aldrich, p.a.), potassium chloride (Merck, p.a.), potassium bromide (Merck, 99%), and hydrochloric acid (Merck, 37%). All solutions were prepared using ultrapure water (18.2 MΩ cm) obtained from an ELGA Lab Water Purelab system. Phosphate buffer solution (PBS, pH = 7) was a mixture 0.15 M K_2_HPO_4_ + 0.10 M KH_2_PO_4_.

Electrochemical experiments were performed in conventional electrochemical glass cells. The working electrode was a glassy carbon rod (geometric area = 0.07 cm^2^, GC, Carbone Lorraine, model V-25). The GC electrode was carefully polished with emery paper and subsequently rinsed with distilled water. A platinum wire was employed as counter electrode, and a reversible hydrogen electrode introduced in the same electrolyte solution in Luggin capillary was used as reference electrode. Cyclic voltammograms were performed with a potentiostat eDAQ EA161 and a digital recorder (eDAQ, ED401) with eDAQ EChart data acquisition software.

The precursor solution to synthesize silica was made by mixing 6 mL TEOS, 8.2 mL EtOH, and 5.8 mL of a solution combining 0.01 M HCl and 0.46 M KCl (the salt was added to provide conductivity). The mixture was magnetically stirred for one hour in a closed vial.

Silica films can be obtained on glassy carbon (GC) surfaces by the following electrochemical procedure. After immersion of the GC into the precursor solution, a negative current was applied to trigger the reduction of water (*j* = –2.5 mA cm^−2^, *t* = 60 s). The increase of pH nearby the electrode activates the gelification of silica, which appears deposited on the electrode surface [19].

Silica morphology was determined by field emission scanning electron microscopy (FESEM, ZEISS model Merlin VP Compact). The silica films were prepared on glassy carbon electrodes and free of moisture, were introduced into the microscope holder without further pretreatment for FESEM analyses. TEM analyses (transmission electron microscopy JEOL JEM-1400 Plus model) were performed on silica samples that were scratched from the surface of GC electrode. The obtained powder was suspended in ethanol and supported on a carbon or metal grid to be studied.

Thermogravimetric analysis (TGA) was performed using a METTLER TOLEDO model TGA/SDTA851e/LF/1600 (Mettler-Toledo AG, Analytical, Schwerzenbach, Switzerland) coupled to a mass spectrometer PFEIFFER VACUUM model THERMOSTAR GSD301T. The silica was collected and dried overnight using a vacuum oven at 65 °C to remove moisture. Thereafter, 20 mg of sample was placed in an alumina crucible and TGA were performed under synthetic atmosphere N_2_:O_2_ (ratio 4:1) with a 100 mL min^−1^ flux, an initial isothermal stage at 25 °C for 120 min and then a temperature ramp of 10 °C min^−1^.

Infrared Spectroscopy was performed using a FTIR, Nicolet 5700 spectrophotometer (Thermo Electron Scientific Instruments, Madison, WI, USA). The collected silica was dried overnight using a vacuum oven at 65 °C to remove moisture. The silica powder was mixed with dry KBr at a concentration of 0.2% w/w to form a pellet for transmission measurements. Spectra were obtained by acquiring 100 interferograms at 8 cm^−1^ resolution.

## 3. Results and Discussion

### 3.1. Characterization of Silica Films

**Figure 1:**
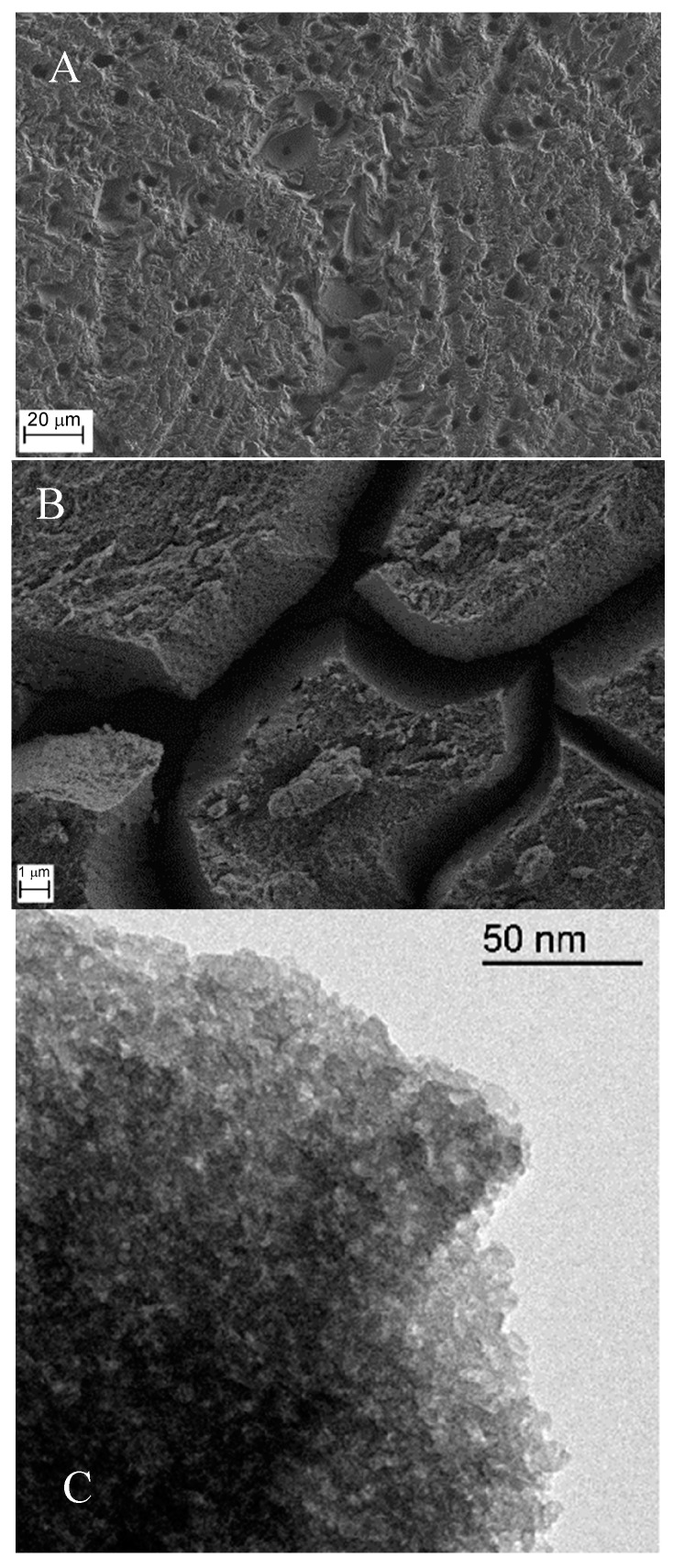
Micrographs obtained by FESEM for an unmodified GC electrode (**A**), a silica-modified electrode (**B**), and a TEM micrograph of the same silica deposit (**C**).

A morphological characterization of the silica layer was performed by field-emission scanning (FESEM) and transmission electron (TEM) microscopies. Figure 1A shows the surface of an unmodified GC electrode as observed by FESEM.

The prevalence of holes and grooves resulting from the polishing procedure gave the surface a heterogeneous character. The silica-modified glassy carbon electrode is shown in Figure 1B, where the silica deposit looks constituted by three dimensional structures of approximately 5 µm in size and 2 μm in thickness, which are promoted by the drying procedure. The TEM micrograph shown in Figure 1C reveals a compact structure resulting from the aggregation of silica colloids of ~5–10 nm in size.

**Figure 2:**
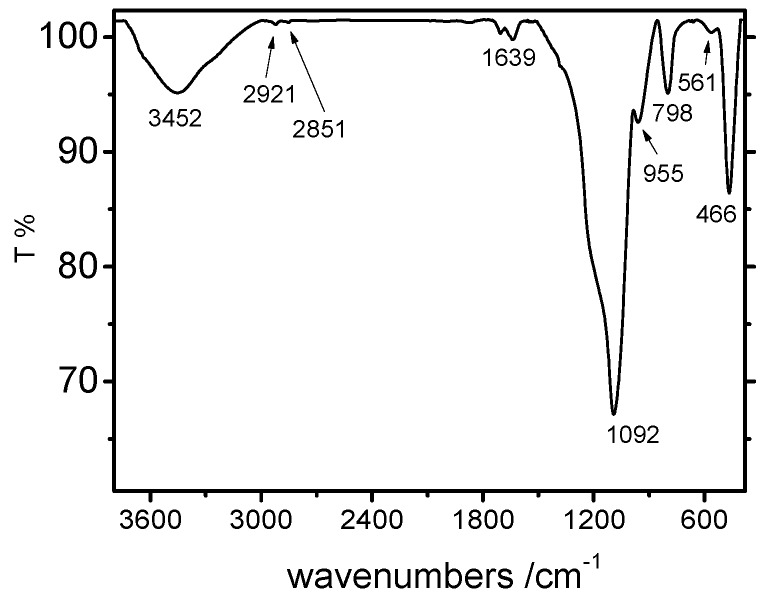
FTIR spectrum of a silica film synthetized by electrochemical methods (100 interferograms, resolution 8 cm^−1^).

Figure 2 shows the FTIR spectrum of a silica sample synthesized by the electrochemical method. The wide absorption band centered at around 3450 cm^−1^ and the associated feature at 1639 cm^−1^ are assigned to OH stretching vibrations and bending vibrations, respectively [20]. Both features come from two different contributions and reveal, on the one hand, the presence of silanol groups (Si–OH) in the silica material and, on the other, the presence of water molecules adsorbed at the gel surface [21]. Surface silanol groups provide a hydrophilic character to silica, which is at the origin of the water signal. Weak bands appearing at 2921 and 2851 cm^−1^ correspond to CH symmetric and antisymmetric stretching modes of aliphatic groups, respectively. They are attributed to the presence of residual ethoxy structural units (CH_3_CH_2_O–) within the gel coming from the uncomplete hydrolyzation of TEOS, the silica precursor.

The band at 955 cm^−1^ can be assigned to the stretching vibration of silanol species [22], but it has been also related to the presence of unhydrolyzed TEOS [23]. Besides, the wide band appearing at 1092 cm^−1^, can be assigned to the antisymmetric stretching vibration of the Si–O–Si structure whereas the symmetric stretching vibration of the same group appears at 798 cm^−1^ [21]. Finally, the band at 466 cm^−1^ corresponds to the bending of the Si–O–Si structure [24] and the band at 561 cm^−1^ to strain defects in the SiO_2_ network [21].

**Figure 3:**
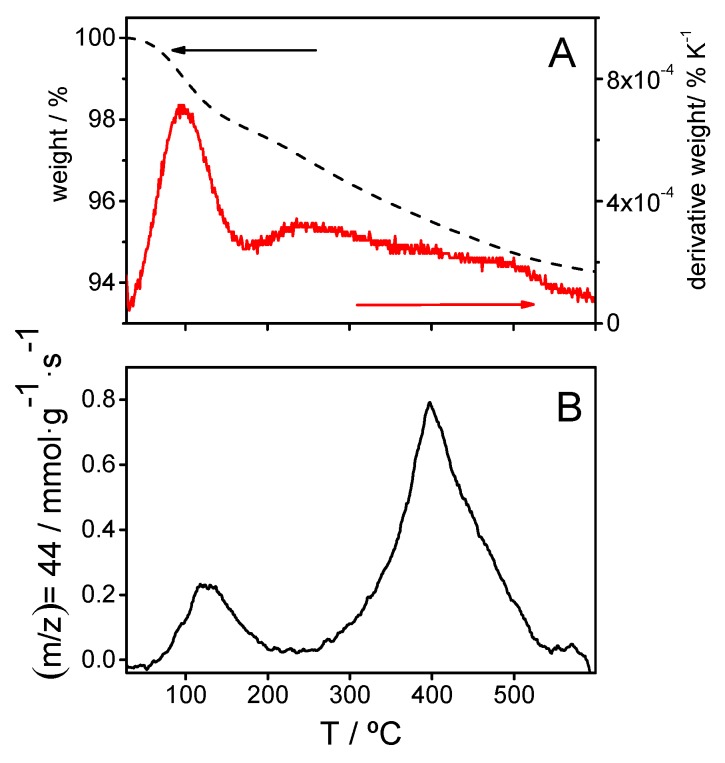
(**A**) Thermogravimetric curve (black dashed line) and first derivative TG curve (red solid line) and (**B**) mass spectrometry signal for m/z = 44 obtained from silica synthetized by the electrochemical method.

To quantify the amount of different species present in the synthesized material, a thermogravimetric analysis of the silica sample was carried out. Figure 3A shows both absolute and differential thermogravimetric curves, where it can be observed that the mass of sample drops from 25 °C and a main feature appears peaking at 100 °C. This latter process is related to the releasing of weakly adsorbed water or ethanol molecules coming from the precursor solvent at the gel pores [25,26]. The mass associated to this process amounts to 27.4 mg per gram of gel.

On the other hand, Figure 3B shows the m/z 44 signal recorded at the mass spectrometer coupled to the TG device. Such feature corresponds to CO_2_ molecules discharged during the thermal treatment at increasing temperatures. A maximum appears at 125 °C corresponding to carbon dioxide coming from the oxidation of adsorbed ethanol, a component of the silica precursor solution. The integration of this signal yields 270 μmol of released CO_2_ per gram of silica sample or, correspondingly, to a content of 6.2 mg ethanol g^−1^. From the total weight loss up to a temperature of 220 °C (27.4 mg g^−1^) the amount of water entrapped into the silica gel can be derived: 21.2 mg g^−1^.

After the complete release of solvents, the TG curve in Figure 3A shows a continuous and featureless weight decay at increasing temperatures, from 220 °C up to 600 °C. From that point, the mass stabilizes at 943 mg per g of initial sample, which means a total weight loss close to 57 mg g^−1^. In parallel, the mass spectrometry scan shows a CO_2_ signal peaking at 395 °C, which comes mainly from the evolution of nonhydrolyzed ethoxy groups covalently linked to silicon atoms [25]. The presence of ethoxy groups was already suggested by the FTIR spectrum in Figure 2. From the integration of the CO_2_ signal (720 μmol g^−1^) the mass of ethoxy groups present initially within the gel, 0.016 mg g^−1^, can be easily obtained assuming the oxidation reaction expressed in Equation (1).

SiO_2−x_(OCH_2_CH_3_)_2x_ (s) + 6x O_2_ (g) → SiO_2_ (s) + 4x CO_2_ (g) + 5x H_2_O (g) (1)

However, some additional contribution different from adsorbed solvents is required to explain the weight loss, approaching 30 mg g^−1^, found in Figure 3A within the 220 to 600 °C temperature range. Such a contribution is the thermal conversion of silanol-functionalized pores into SiO_2_, as shown in Equation (2).

SiO_2−y_(OH)_2y_ (s) + 0.5y O_2_ (g) → SiO_2_ (s) + 2y H_2_O (g) (2)

Combining both chemical reactions, the empirical formula of silica gel prepared by electrochemical deposition can be determined: SiO_1.963_(OH)_0.051_(OCH_2_CH_3_)_0.022_. This result suggests that, roughly, one in 14 silicon atoms are oriented towards gel pores, which are functionalized mostly with silanol centers and, to a lesser degree, with ethoxy groups. The surface area obtained from the BET (Brunauer–Emmett–Teller theory) analysis of electrodeposited silica was measured in a previous contribution [27] and found to be 445 m^2^ g^−1^. From that result, a surface concentration of functionalized groups (both ethoxy and silanol) close to 1.6 sites nm^−2^ can be derived, in good agreement with data previously published for silica gels [28]. The mass of water adsorbed at silica can be also expressed in terms of relative surface concentration as 0.073 mol H_2_O/mol Si or, equivalently, as 1.6 H_2_O molecules nm^−2^, a figure that fits well with the fraction of functionalized sites in silica pores (resulting $\theta_{H_{2}O}\sim 1$). That value strongly suggests that water molecules are effectively coordinated with surface groups.

### 3.2. Electrochemical Behavior of Neurotransmitters on Bare GC Surfaces

**Figure 4:**
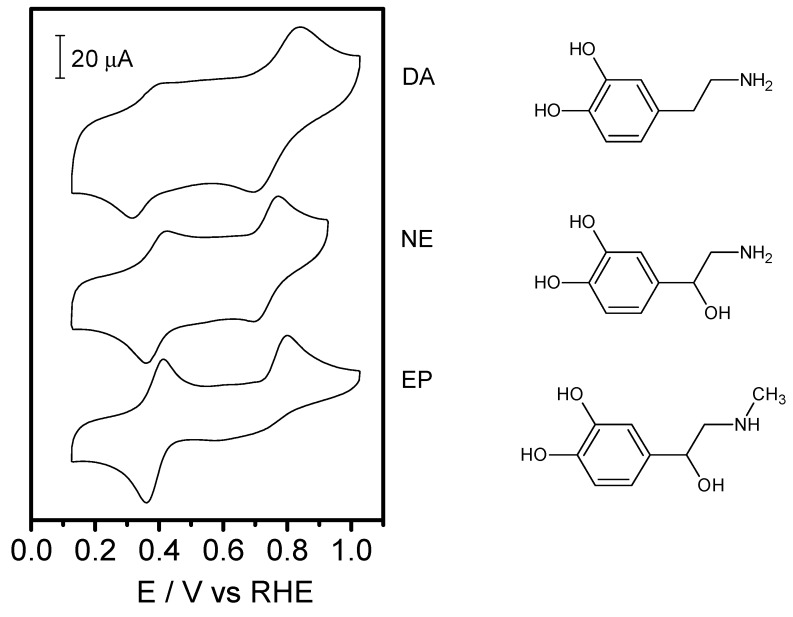
Stabilized cyclic voltammograms for a glassy carbon electrode immersed in in phosphate buffer solution (PBS) solutions (pH 7) containing 1 mM neurotransmitter: dopamine (DA), norepinephrine (NE), and epinephrine (EP). Scan rate 100 mVs^−1^.

As a previous step to analyze the affinity of silica material for neurotransmitters (NT), it is necessary to characterize the electrochemical response of dopamine, epinephrine and norepinephrine separately on bare electrodes, i.e., with no silica layer deposited. Figure 4 shows the stabilized voltammetric profiles recorded with a bare GC electrode immersed in PBS solutions containing 1 mM of each NT.

The recorded cyclic voltammetry (CV) for dopamine shows a main oxidation peak at 0.84 V during the forward scan. Similar oxidation responses are observed for norepinephrine and epinephrine (peaking at 0.78 and 0.80 V, respectively). Those features correspond to the oxidation of the molecules to the amine-*o*-quinone forms (AoQ).

**Scheme 1:**
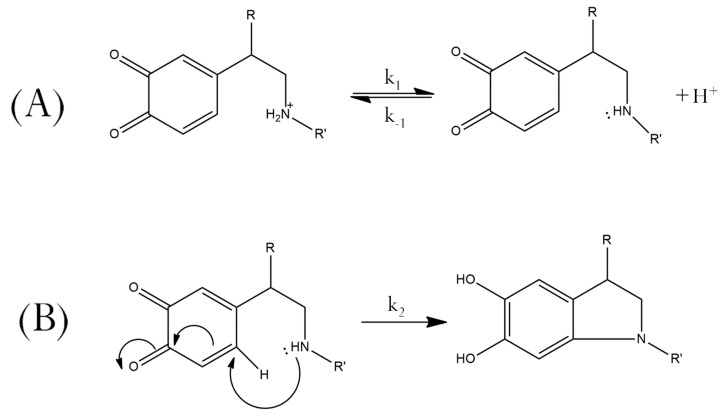
Intermediate chemical stage of the ECE mechanism widely-accepted for the oxidation of catecholamines. (**A**) *k_1_* and *k_−1_* correspond to the kinetic constants for the protonation of the amine group (related to the acid dissociation constant, *K_a_*). (**B**) The unprotonated amine-*o*-quinone (AoQ) undergoes cyclization with a characteristic kinetic constant, *k_2_*.

After electrochemical oxidation, these AoQ may undergo a subsequent chemical reaction characterized by an inner nucleophilic attack of the amine group to the quinone ring. This mechanism is known as ECE (see Scheme 1) [29,30,31].

After the chemical step, the cyclized products (leucodopaminochrome for the case of dopamine and equivalent compounds for the rest of neurotransmitters) suffer redox transitions appearing in the CV as a pair of peaks at potentials below 0.4 V.

Particularly, the redox processes involving the parent compounds and the cyclized products appear at similar potential values. Some subtle differences in the intensity of redox peaks can be observed. On the one hand, cathodic peaks related to the reduction of AoQ species are slightly less intense and defined for NE and EP. On the other, those peaks corresponding to the redox transitions of cyclized compounds appear more intense and defined for NE and EP than for DA. These differences can be explained in terms of the concentration of species suffering cyclization, which is only the unprotonated AoQ (see Scheme 1). The protonation level is determined by the p*K_a_* value of the respective amine groups, AoQH^+^, which are 10.6 (DA), 9.8 (NE), and 9.9 (EP) [32]. So, the concentration of AoQ species available to undergo cyclization in neutral PBS solution is higher for NE and EP and the intensity of their low potential voltammetric peaks rises correspondingly.

### 3.3. Characterization of the Adsorption of Neurotransmitters at Silica Films

**Figure 5:**
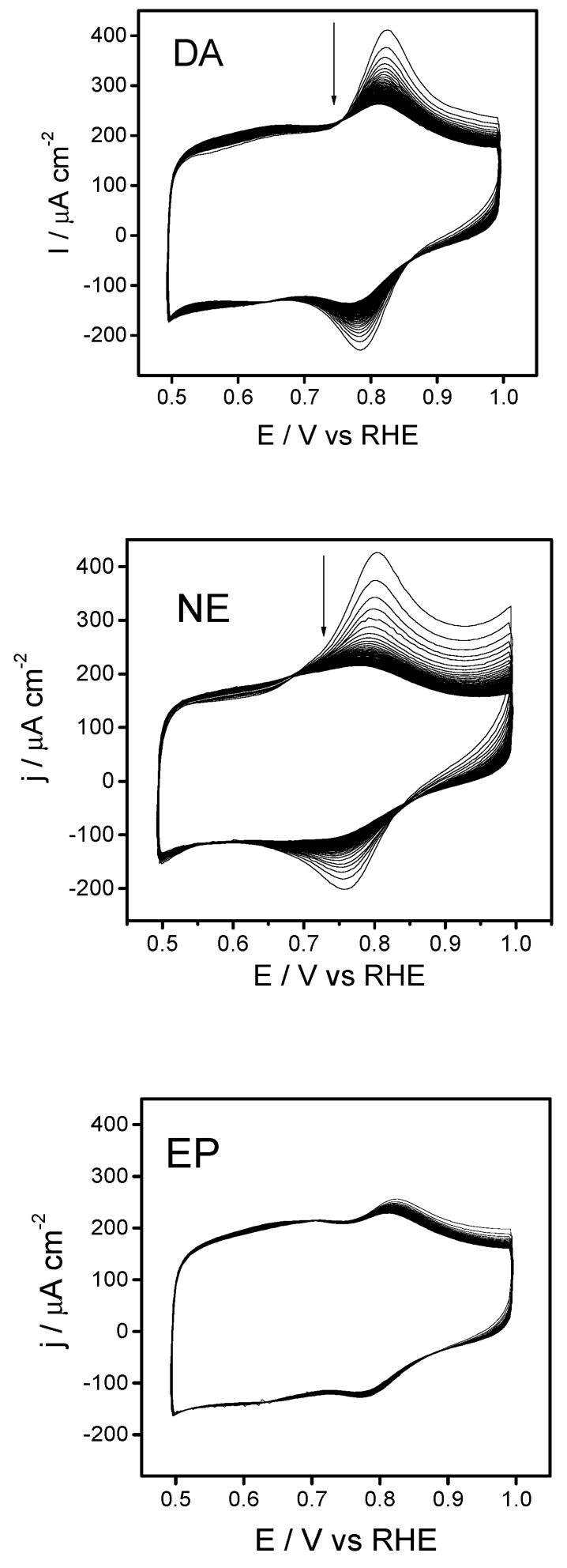
Successive cyclic voltammograms recorded for a silica-modified GC electrode recorded in a blank PBS solution (pH 7) after equilibration for 10 min in either 1mM DA, 1 mM NE, or 1mM EP solution. T = 25 °C. Scan rate 100 mV s^−1^ in all cases.

Solid–liquid adsorption processes are characterized by the occurrence of an interaction between sorbate molecules contained in the liquid phase and the solid sorbent material. The affinity of the sorbent for sorbate molecules determines their distribution between both phases once the equilibrium has been reached. Owing to the physicochemical characteristics of silica films, it is expected that this material shows high enough affinity for neurotransmitters to produce their significant retention at the solid surface. To check this, the adsorption of catecholamine neurotransmitters has been investigated and compared in the present section. A GC electrode covered by a freshly deposited silica layer was immersed in 1 mM neurotransmitter solution prepared in PBS electrolyte (pH 7). The contact was maintained for 10 min to allow the permeation of analyte molecules through silica pores and their interaction with the surface. The selection of this particular contact time will be justified below. After this step, the modified electrode loaded with the corresponding neurotransmitter was rinsed and transferred to an electrochemical cell containing PBS (blank solution). Figure 5 shows successive cyclic voltammograms recorded for each NT immediately after immersion in the blank solution.

For dopamine, the first forward scan shows an anodic peak at 0.84 V revealing the oxidation of adsorbed DA to its AoQ, dopaminequinone. In the reverse scan, a counter process ascribed to the reduction of AoQ can be clearly observed. The anodic peak intensity decreases during the subsequent potential cycles and vanishes after ~35 scans, an observation that suggests the neurotransmitter is being released to the bulk solution. Parallel voltammetric responses are recorded for norepinephrine and epinephrine although this latter species shows lower peak currents.

**Figure 6:**
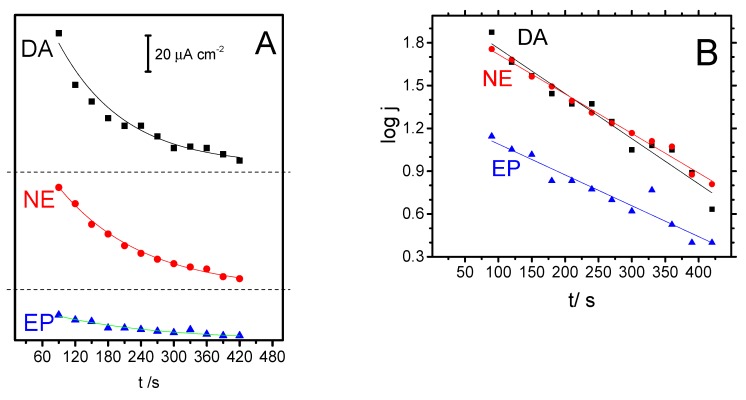
(**A**) Peak current density for catecholamine neurotransmitters (NT) oxidation in blank PBS solution after equilibration in 1 mM NT solution. Symbols denote experimental data, while the solid curves have been obtained by fitting a first-order decay and (**B**) logarithmic time dependence of the oxidation peak current density for each NT.

In the absence of adsorbed products that could block the electrode active sites of the glassy carbon, cyclic voltammetry curves show typically peak currents which are proportional to analyte concentration [33]. This statement can be also applied to Figure 5, provided that proportionality is limited to the apparent concentration of DA in the vicinity of the GC surface. The restriction applies because active molecules are continuously released to the bulk solution from silica pores, as demonstrated by Figure 6A. There, plots of peak current density (extracted from Figure 5) against the length of the experiment reveals the current drop associated to the depletion of NT. The experimental points can be well described by first-order decays (Equation (3)), which are represented by solid lines in Figure 6A.

(3) $$j=j_{0}e^{-kt}$$

where *j_0_* is the peak current density corresponding to the oxidation of the initial concentration of NT present inside the silica film and *k* is a pseudo first-order rate constant for the desorption process. The logarithmic graphs of data obtained from Figure 6A are depicted in Figure 6B, for which good correlations exist and, consequently, values for *k* and *j_0_* parameters can be obtained from a line fitting (see Table 1).

Owing to its electrochemical nature, extrapolated *j_0_* currents can be used to determine the apparent concentration of neurotransmitter trapped inside the silica film, ${\left[NT\right]}_{gel}^{}$, employing the Randles–Sevcik Equation [33]:

(4) $$j_{0}=\left(2.69\cdot 10^{5}\right)n^{3/2}D_{}^{1/2}{\left[NT\right]}_{gel}^{}v^{1/2}$$

with *n* the number of electrons transferred (2 for the oxidation of each NT to its AoQ form), *D* the diffusion coefficient of neurotransmitter molecules in cm^2^ s^−1^ (see Table 1), and *υ* the scan rate in V s^−1^.

The apparent concentrations of dopamine and norepinephrine derived from Equation (4) are 82.8 and 74.7 mM, respectively. Both values are almost two orders of magnitude above the concentration employed in the equilibration solutions ([NT] = 1.0 mM). However, for epinephrine, the apparent concentration inside the gel is only one order of magnitude larger than in the equilibration solution (16.7 mM). These results reveal that silica exhibits a significant affinity for neurotransmitters and consequently the solid phase is acting as an effective preconcentration layer for these molecules. At this point it is worth mentioning that the time required to reach the adsorption equilibrium (the equilibration time) was determined for each neurotransmitter by representing diagrams of apparent concentration, ${\left[NT\right]}_{gel}^{}$, at increasing adsorption times (see Figure S1 in the supporting information). It was observed that a 10-min adsorption period is enough to reach equilibrium under the experimental conditions employed.

**Figure 7:**
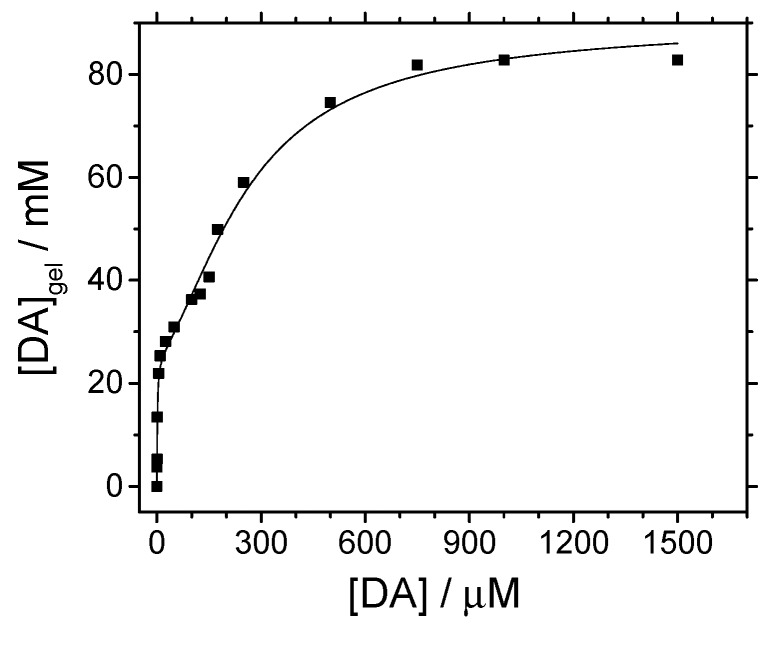

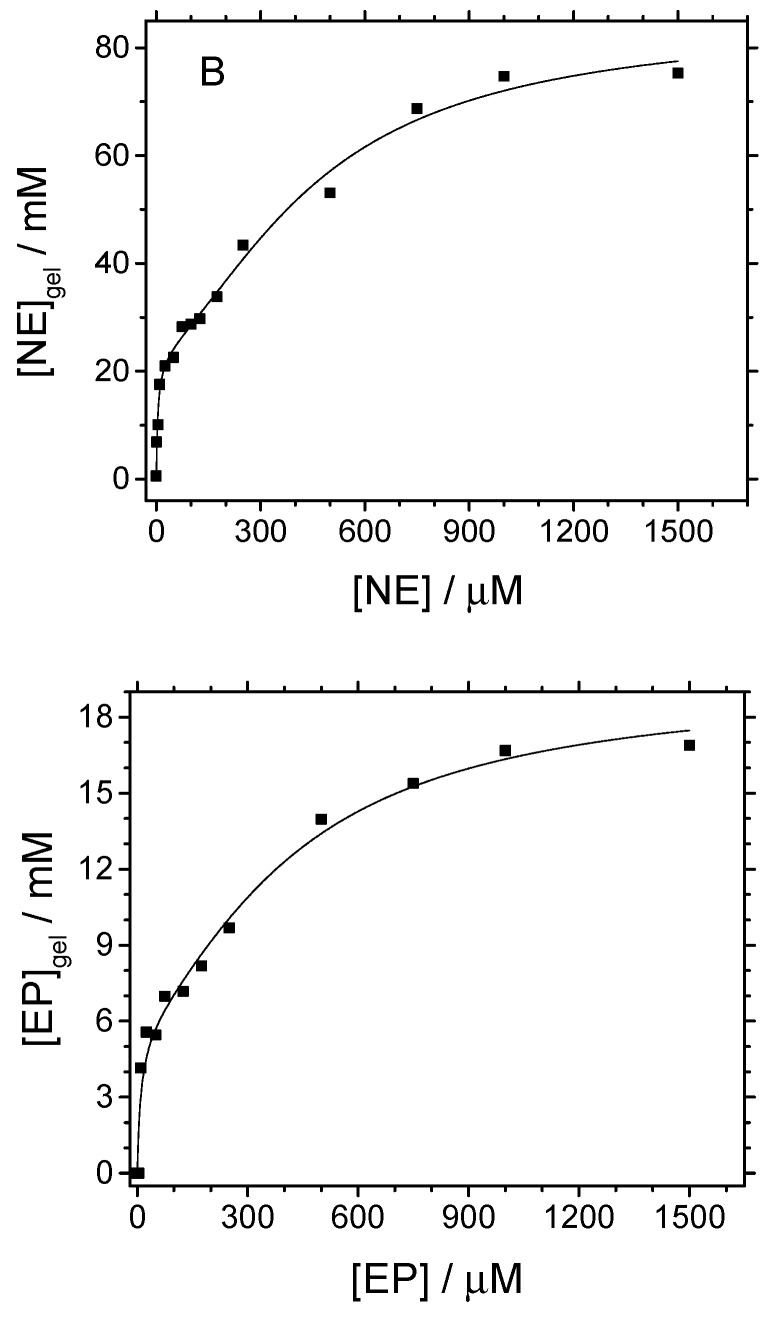
Neurotransmitters adsorption isotherms determined for silica-modified GC electrodes in PBS solutions, pH 7. Equilibration time was 10 min in all cases. T = 25 °C.

The adsorption capacity of silica can be determined for each neurotransmitter by equilibrating the film at increasing concentration of NT and then measuring the trapped amount (expressed in terms of apparent concentration,$\;{\left[NT\right]}_{gel}^{}$). Such an experiment is shown in Figure 7, where the obtained graphs correspond to solid–liquid adsorption isotherms of NT.

Shape of curves in Figure 7 fit well with L-type isotherms, subgroup 4, according to the classification of Giles et al. [35]. This means that a monolayer of NT adsorbs first over silica sites and then multilayer adsorption occurs. For low concentrated equilibration solutions, the apparent concentration of NT obtained from CV results (the amount of adsorbed species) increases almost linearly. Similar isotherms were obtained for the adsorption process of other polar organic molecules such as benzyl alcohol, benzaldehyde, or benzoic acid on silica [28,36]. As deduced from the onset of the first adsorption plateau, the monolayer completion is reached at ~15 *μ*M for the three NT. For dopamine, the multilayer adsorption regime starts at [DA] ~160 *μ*M and extends up to a second plateau appearing at 800 µM, which represents the saturation of accessible sites. The onset of multilayer adsorption is very similar for the other two neurotransmitters, [NE] ~140 *μ*M and [EP] ~150 *μ*M. However, the saturation of adsorption sites takes place at a higher concentration, of ~1000 µM, in both cases.

Experimental points in Figure 7 can be fitted to the BET isotherm for liquid phase adsorption (Equation (5)) as proposed by Ebadi et al. [37]:

(5) $${\left[NT\right]}_{gel}={\left[NT\right]}_{m}\frac{k_{m}\left[NT\right]\left[1-\left(n+1\right){\left(k_{L}\left[NT\right]\right)}^{n}+n{\left(k_{L}\left[NT\right]\right)}^{n+1}\right]}{\left(1-k_{L}\left[NT\right]\right)\left[1+\left(\frac{k_{m}}{k_{L}}-1\right)k_{L}\left[NT\right]-\left(\frac{k_{m}}{k_{L}}\right){\left(k_{L}\left[NT\right]\right)}^{n+1}\right]}$$

In this expression, ${\left[NT\right]}_{m}$ represents the apparent concentration of neurotransmitter equivalent to an adsorbed monolayer, *k_m_* is the affinity constant for the formation of a monolayer (a measure of the affinity of silica for the neurotransmitter), *n* the maximum number of adsorbed layers, and *k_L_* the affinity constant for multilayers. The parameters derived from this isotherm have been summarized in Table 2.

According to the modified BET model, a monolayer of dopamine represents an apparent concentration close to 25 mM within the silica. Hence, taking the silica hydrogel density as 1.0 g cm^−3^ [21,38] and its surface area 445 m^2^ g^−1^, as determined by BET analysis, it is derived that a monolayer is constituted by a surface density close to 0.034 DA molecules nm^−2^. Such a value corresponds to a surface coverage close to $\theta_{DA}$= 1.4 %. Similar result is obtained for norepinephrine (see Table 2). Epinephrine neurotransmitter gives a smaller value (by a factor of 4) for the apparent concentration of the adsorbed monolayer.

An analysis of the relative values of affinity constants for the formation of monolayers shows that NE and EP present lower *k_m_* than DA by factors of 4 and 10, respectively, which strongly suggests weaker interaction with the silica surface. *k_m_* values are closely related to the adsorption process of the *NT* on silica sites, as represented in Equation (6)

*NT_(aq)_* + *adsorption site* ⇌ *NT_(ads)_* (6)

The characteristic equilibrium constant for this process, *K_m_*, is numerically equivalent to *k_m_*, the affinity constant derived from Equation (5). This thermodynamic constant, *K_m_*, can be related to the free energy of adsorption of the monolayer, Δ*G^o^* (*NT_m_*), a measure of the strength of the interaction between neurotransmitters and silica:

(7) $$K_{m}=e^{-\frac{\Delta G^{0}\left(NT_{m}\right)}{RT}}$$

Therefore, by comparing *K_m_* values we can determine differences in the adsorption energy of neurotransmitters. For DA and EP,

(8) $$ln\frac{K_{m}{\left(DA_{m}\right)}_{1}}{K_{m}{\left(EP_{m}\right)}_{2}}=\frac{\Delta G^{0}{\left(DA_{m}\right)}_{2}-\Delta G^{0}{\left(EP_{m}\right)}_{1}}{RT}$$

As derived from Equation (8), the free energy of adsorption of EP is 5.5 kJ mol^−1^ more negative than that of DA. Assuming similar entropy changes during the adsorption process, the major contribution to the free energy difference should be a disparity in the enthalpy of the adsorption process. Catecholamine neurotransmitters have similar chemical structures that could hardly justify the observed differences, except if hydrogen bonding is considered [28,36]. It should be noted that DA and NE are both primary amines presenting similar surface coverage for the adsorbed monolayer. It can be then inferred that the existence of an additional hydroxyl group at the aliphatic chain of neurotransmitters has little effect on the adsorption process. On the other hand, the chemical structure of EP looks equivalent to that of NE (regarding the presence of the same aliphatic –OH group) but it contains a secondary amine, which could be at the origin of the significantly lower surface coverage obtained for this particular NT. In the light of this interpretation, differences in adsorption energies reveal the prevalence of hydrogen bonding between silanol groups located at the silica surface and amine groups, either primary or secondary, of neurotransmitters. Similar energy differences were reported in the literature, where the enthalpy of formation of hydrogen bonds between alcohols and primary amines was 5 to 20 kJ mol^−1^ more negative than that involving secondary amines [39,40], in agreement with our results.

## 4. Conclusions

Glassy carbon electrodes have been modified with homogeneous silica films synthesized by electrochemical methods using a sol–gel precursor solution. FTIR experiments confirmed the presence of functionalized Si centers in the silica material, mainly in the form of silanol groups but with a minor amount of residual ethoxy groups coming from the uncomplete hydrolyzation of TEOS, the silica precursor. Based on TG results, an empirical formula for electrodeposited silica gel was proposed, for which it was estimated that approximately one in 14 functionalized (hydrophilic) silicon atoms are oriented towards the solid–liquid interface.

Silica films show high affinity for dopamine, norepinephrine, and epinephrine, causing a significant retention of these molecules. The adsorption capacity was determined for each neurotransmitter by means of solid–liquid adsorption isotherms which, according to the classification of Giles, can be classified as L-type isotherms (subgroup 4) in all cases. It was derived that the surface packing density of DA and NE monolayers approach 0.034 molecules nm^−2^, corresponding to a coverage close to 1.4%. In contrast, the monolayer of adsorbed epinephrine restricts to 0.03% surface coverage.

Contrary to dopamine and norepinephrine, epinephrine is a secondary amine, which, according to the free energy differences estimated for the adsorption process, strongly suggests that hydrogen bonds between the amine group of neurotransmitters and silanol groups at silica is the main adsorption mechanism. However, the existence of minor interactions between alcohol groups of neurotransmitters and surface silanols cannot be ruled out.

In summary, electrodes modified with silica films can be easily prepared, they are chemically stable and show different interaction with the studied neurotransmitters. Consequently, they present potential applications as electrochemical sensors for the selective detection of these biologically active molecules.

## Figures and Tables

**Table 1:** Pseudo first-order rate constants for the desorption of neurotransmitters, initial anodic peak current density, diffusion coefficient [34] and apparent concentration of adsorbed neurotransmitters obtained from Equation (4).

Parameter	DA	NE	EP
k/s^−1^	7.30 × 10^−3^	6.38 × 10^−3^	4.97 × 10^−3^
$j_{0}$/μA cm^−2^	120	98.8	20.3
*D*/cm^2^ s^−1^	6.0 × 10^−6^	5.2 × 10^−6^	5.5 × 10^−6^
${\left[NT\right]}_{gel}^{}$/mM	82.8	74.7	16.7

**Table 2:** Calculated parameters for the adsorption isotherms of neurotransmitters: concentration of neurotransmitter equivalent to an adsorbed monolayer ${\left[NT\right]}_{m}$, affinity constant for the formation of a monolayer $k_{m}$, affinity constant for multilayers $k_{L}$, maximum number of adsorbed layers *n*, and surface coverage $\theta_{NT}$.

Parameter	DA	NE	EP
${\left[NT\right]}_{m}$/ mM	24.8	23.4	6.0
$k_{m}$/ mM^−1^	863	209	94
$k_{L}$/ mM^−1^	3.8	2.2	2.3
n	4	4	3
$\theta_{NT}$/ %	1.4	1.3	0.3

## Supplementary Materials

The apparent concentration of different neurotransmitter in the silica gel electrode as function of the incubation time (Figure S1) are available online at http://www.mdpi.com/1424-8220/19/4/868/s1.
