# Supplementary material for: Affinity of Electrochemically Deposited Sol–Gel Silica Films towards Catecholamine Neurotransmitters
Source: Sensors (Basel). 2019 Feb 19;19(4):868. doi: 10.3390/s19040868 (PMC6412732; doi:10.3390/s19040868)
Supplement: Supplementary file 1 [file sensors-19-00868-s001.pdf]

Supporting information for

Affinity of Electrochemically Deposited Sol–Gel Silica Films Towards Catecholamine Neurotransmitters

María Porcel-Valenzuela [1], Francisco Huerta [2], Emilia Morallón [1], Francisco Montilla [1,*]

[1] Departmento Química Física e Instituto Universitario de Materiales, Universidad de Alicante, Carretera San Vicente del Raspeig s/n San Vicente del Raspeig, Alicante E-03690, Spain; maria.porcel@ua.es (M.P.-V.); morallon@ua.es (E.M.)

[2] Departmento Ingenieria Textil y Papelera, Universitat Politecnica de Valencia, Plaza Ferrandiz y Carbonell 1, Alcoy E-03801, Spain; frahuear@txp.upv.es

* Correspondence: francisco.montilla@ua.es

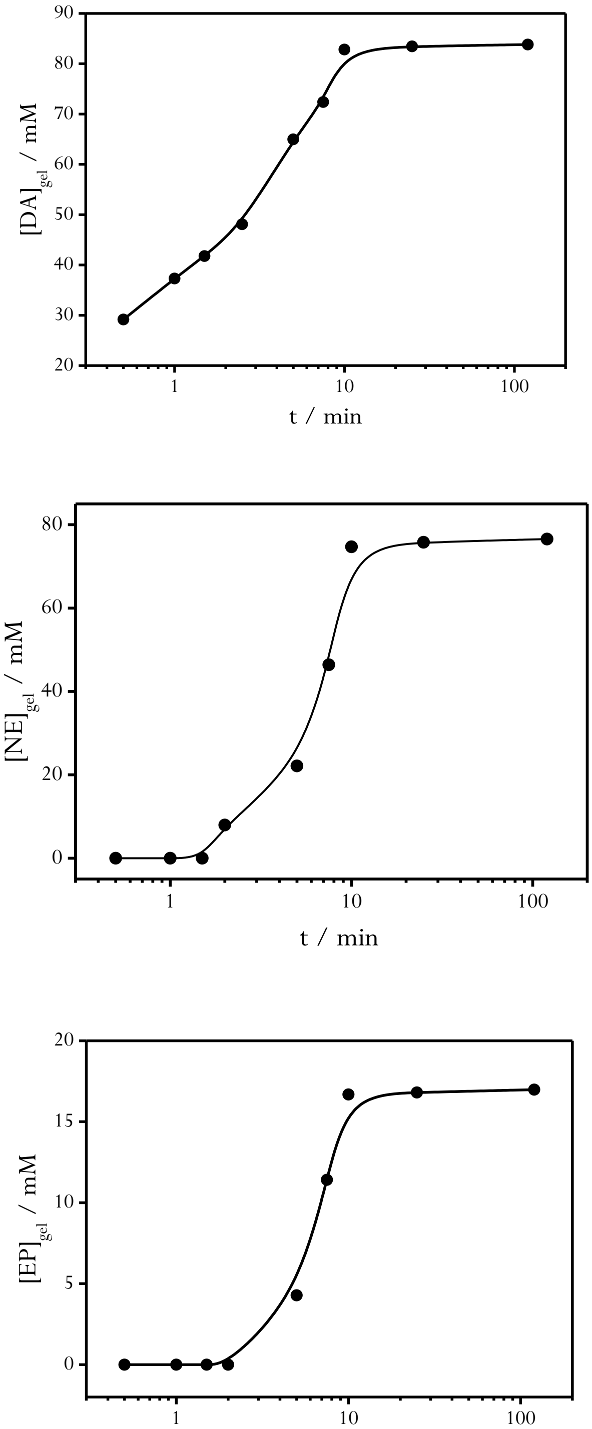

Figure S1: Apparent concentration of different neurotransmitter in the silica gel electrode as function of the incubation time in PBS pH 7 (A: 1 mM DA; B: 1 mM NE; C: 1 mM EP).
